# Evidence for a Causal Role for Escherichia coli Strains Identified as Adherent-Invasive (AIEC) in Intestinal Inflammation

**DOI:** 10.1128/msphere.00478-22

**Published:** 2023-03-08

**Authors:** Hatem Kittana, João C. Gomes-Neto, Kari Heck, Anthony F. Juritsch, Jason Sughroue, Yibo Xian, Sara Mantz, Rafael R. Segura Muñoz, Liz A. Cody, Robert J. Schmaltz, Christopher L. Anderson, Rodney A. Moxley, Jesse M. Hostetter, Samodha C. Fernando, Jennifer Clarke, Stephen D. Kachman, Clayton E. Cressler, Andrew K. Benson, Jens Walter, Amanda E. Ramer-Tait

**Affiliations:** a Department of Food Science and Technology, University of Nebraska-Lincoln, Lincoln, Nebraska, USA; b Department of Animal Science, University of Nebraska-Lincoln, Lincoln, Nebraska, USA; c School of Veterinary Medicine and Biomedical Sciences, University of Nebraska-Lincoln, Lincoln, Nebraska, USA; d Department of Veterinary Pathology, College of Veterinary Medicine, Iowa State University, Ames, Iowa, USA; e Department of Pathology, University of Georgia, Athens, Georgia, USA; f Department of Statistics, University of Nebraska-Lincoln, Lincoln, Nebraska, USA; g Nebraska Food for Health Center, University of Nebraska-Lincoln, Lincoln, Nebraska, USA; h School of Biological Sciences, University of Nebraska-Lincoln, Lincoln, Nebraska, USA; i Department of Food and Nutritional Science, University of Alberta, Edmonton, Alberta, Canada; j Department of Biological Sciences, University of Alberta, Edmonton, Alberta, Canada; k APC Microbiome Ireland, School of Microbiology and Department of Medicine, University College Cork-National University of Ireland, Cork, Ireland; University of Kentucky

**Keywords:** inflammatory bowel disease, adherent-invasive *E. coli*, macrophages, epithelial cells, gnotobiotic mice

## Abstract

Enrichment of adherent-invasive Escherichia coli (AIEC) has been consistently detected in subsets of inflammatory bowel disease (IBD) patients. Although some AIEC strains cause colitis in animal models, these studies did not systematically compare AIEC with non-AIEC strains, and causal links between AIEC and disease are still disputed. Specifically, it remains unclear whether AIEC shows enhanced pathogenicity compared to that of commensal E. coli found in the same ecological microhabitat and if the *in vitro* phenotypes used to classify strains as AIEC are pathologically relevant. Here, we utilized *in vitro* phenotyping and a murine model of intestinal inflammation to systematically compare strains identified as AIEC with those identified as non-AIEC and relate AIEC phenotypes to pathogenicity. Strains identified as AIEC caused, on average, more severe intestinal inflammation. Intracellular survival/replication phenotypes routinely used to classify AIEC positively correlated with disease, while adherence to epithelial cells and tumor necrosis factor alpha production by macrophages did not. This knowledge was then applied to design and test a strategy to prevent inflammation by selecting E. coli strains that adhered to epithelial cells but poorly survived/replicated intracellularly. Two E. coli strains that ameliorated AIEC-mediated disease were subsequently identified. In summary, our results show a relationship between intracellular survival/replication in E. coli and pathology in murine colitis, suggesting that strains possessing these phenotypes might not only become enriched in human IBD but also contribute to disease. We provide new evidence that specific AIEC phenotypes are pathologically relevant and proof of principle that such mechanistic information can be therapeutically exploited to alleviate intestinal inflammation.

**IMPORTANCE** Inflammatory bowel disease (IBD) is associated with an altered gut microbiota composition, including expansion of *Proteobacteria*. Many species in this phylum are thought to contribute to disease under certain conditions, including adherent-invasive Escherichia coli (AIEC) strains, which are enriched in some patients. However, whether this bloom contributes to disease or is just a response to IBD-associated physiological changes is unknown. Although assigning causality is challenging, appropriate animal models can test the hypothesis that AIEC strains have an enhanced ability to cause colitis in comparison to other gut commensal E. coli strains and to identify bacterial traits contributing to virulence. We observed that AIEC strains are generally more pathogenic than commensal E. coli and that bacterial intracellular survival/replication phenotypes contributed to disease. We also found that E. coli strains lacking primary virulence traits can prevent inflammation. Our findings provide critical information on E. coli pathogenicity that may inform development of IBD diagnostic tools and therapies.

## INTRODUCTION

Clinical, epidemiologic, and animal studies collectively demonstrate the multifaceted nature of inflammatory bowel diseases (IBD), including Crohn’s disease (CD) and ulcerative colitis (UC). These diseases arise from complex interactions between predisposing genetic factors and environmental triggers that induce aberrant immune responses against the gut microbiota (1). Additionally, microbial dysbiosis is a well-documented phenomenon associated with IBD (1, 2). Although it is not yet clear if dysbiosis is a cause or a consequence of inflammation (3), abnormalities in the IBD-associated microbiota include the contraction of taxa known to be beneficial to human health, including *Faecalibacterium*, *Roseburia*, and *Alistipes*, as well as an enrichment of *Pseudomonadota* (4, 5). Importantly, several members of this taxonomic group (including Escherichia coli) have been hypothesized to behave as pathobionts—commensal microorganisms that may contribute to disease processes under select environmental or genetic pressures (6–8).

One group of these proposed pathobionts, adherent-invasive Escherichia coli (AIEC), is selectively enriched in the ileal mucosa in subsets of Crohn’s patients, suggesting that expansion of AIEC populations may occur during inflammation (9–15). Although AIEC strains were first described over 20 years ago (11), our understanding of exactly how these organisms contribute to IBD pathogenesis remains limited (3, 16). AIEC strains lack the known primary virulence factors and invasive determinants of other E. coli pathotypes (17, 18). Moreover, numerous comparative genomics studies have failed to identify virulence genes or molecular properties that are either shared by or exclusive to AIEC (9, 13, 15, 19–26). Consequently, AIEC remains loosely defined by *in vitro* phenotypic characteristics and an absence of the canonical pathogenic determinants found in pathovars of diarrheagenic E. coli.

Glasser and colleagues defined any mucosa-associated E. coli strain as AIEC based solely upon *in vitro* characteristics, including survival and replication inside the mouse macrophage cell line J774, production of high levels of the proinflammatory cytokine tumor necrosis factor alpha (TNF-α) from infected J774 macrophages (27), and adherence and invasion in the human epithelial cell line Caco2 (17). Some isolates classified as AIEC based on these *in vitro* characteristics have been shown to cause colitis in animal models (28–32). However, studies so far have evaluated only one or two strains at a time and have not systematically compared AIEC strains to commensal E. coli to account for strain-to-strain variation. Moreover, several studies have used lab-adapted/modified E. coli strains as controls (28, 33, 34) (e.g., K-12 derivatives) that are not genetically, physiologically, or ecologically comparable to AIEC. Proving Koch’s postulates for AIEC strains has been further complicated by the use of mice harboring a complex microbiota that already includes E. coli (28, 29, 31, 32). Consequently, it is difficult to determine if disease is caused by the AIEC strains being tested or if it includes contributions from resident E. coli. Considering these limitations collectively, it remains disputed if strains classified as AIEC truly have a greater propensity to cause pathology than commensal E. coli found in the same ecological microhabitat (3, 35). Additionally, the extent to which the various *in vitro* phenotypes used to classify strains as AIEC is pathologically relevant has not been systematically tested. Such an evaluation is important given the substantial amount of genetic variation among AIEC strains (15, 36).

The goal of this study was to perform a systematic characterization of 30 human-derived E. coli strains to test the hypothesis that strains displaying an *in vitro* AIEC phenotype are more pathogenic in a murine model of intestinal inflammation than are commensal E. coli strains. We then determined the relevance of the different AIEC phenotypic traits associated with disease in mice. Finally, we explored the possibility of applying this mechanistic information on the pathogenicity of E. coli strains to develop a therapeutic intervention.

## RESULTS

### Mucosa-associated, human-derived E. coli strains are not a monophyletic population.

**FIG 1:**
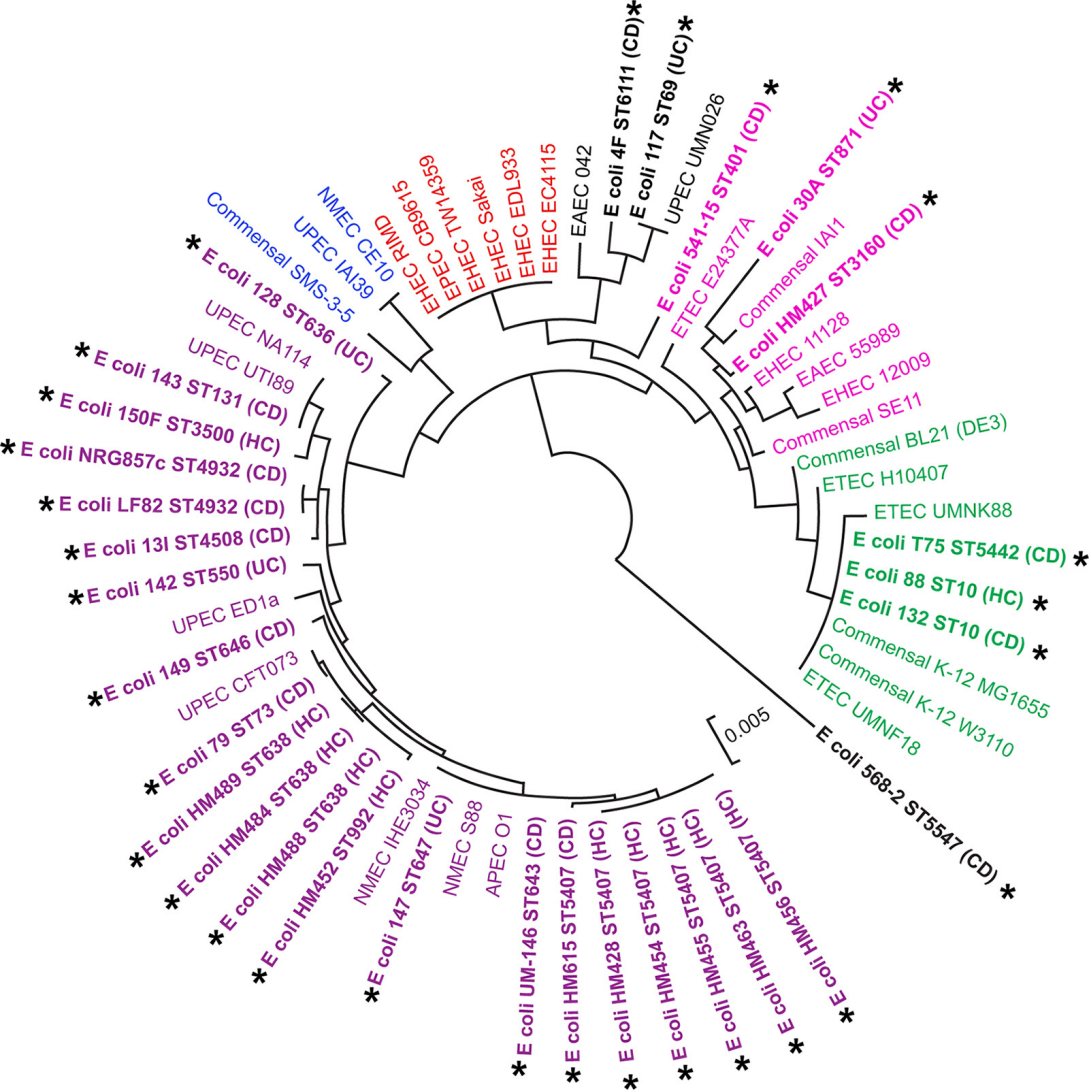
Phylogenetic assignments of the 30 mucosa-associated, human-derived E. coli strains evaluated in this study. A maximum likelihood tree for a total of 60 E. coli strains was constructed using the concatenated nucleotide sequence of seven MLST genes. Each color indicates the phylogenetic group of E. coli (A, green; B1, pink; B2, purple; D, black; E, red; F, blue). The 30 E. coli strains evaluated in this study are in bold and labeled with asterisks. The scale bar represents 0.005 nucleotide substitutions per site.

To systematically relate *in vitro* AIEC phenotypes to *in vivo* pathogenicity, we obtained 30 E. coli strains isolated from the mucosa of either UC or CD patients or healthy individuals reported in previous studies (see Table S1 in the supplemental material). To determine the phylogenetic relatedness of these 30 E. coli strains to different E. coli phylogroups, a maximum likelihood (ML) tree was constructed based upon the concatenated nucleotide sequences of seven housekeeping genes reported by Wirth et al. (37). In addition, multilocus sequencing typing (MLST) sequences of 30 pathogenic and commensal E. coli strains isolated from human intestinal samples were included in the ML tree. These 30 reference E. coli strains were chosen because they represented the six different E. coli phylogroups (A, B1, B2, D, E, and F) (38). The 30 E. coli strains tested in our study were distributed within different E. coli phylogroups and did not represent a distinct phylogenetic clade (Fig. 1). The vast majority of test strains (21 of 30) clustered with E. coli strains belonging to phylogroup B2, which primarily includes pathogenic E. coli isolates that cause extraintestinal infections. These 21 strains were shown to originate from 13 different sequence types (ST), with multiple closely related strains sharing an ST (Table S1). The remaining nine strains were distributed across phylogroups A, B1, and D, and they represented eight different ST. No new ST were identified among the 30 E. coli isolates we tested. Together, these results show that the E. coli strains included in this study are not a monophyletic population but rather represent a heterogeneous population that includes phylogenetically distant strains distributed across all known E. coli phylogroups as others have previously reported (9, 13, 15).

10.1128/msphere.00478-22.4 TABLE S1 Mucosa-associated E. coli strains used in this study. Download Table S1, PDF file, 0.1 MB. Copyright © 2023 Kittana et al. 2023 Kittana et al. https://creativecommons.org/licenses/by/4.0/ This content is distributed under the terms of the Creative Commons Attribution 4.0 International license.

### Substantial variation in *in vitro* AIEC phenotypes exists among mucosa-associated E. coli strains.

**FIG 2:**
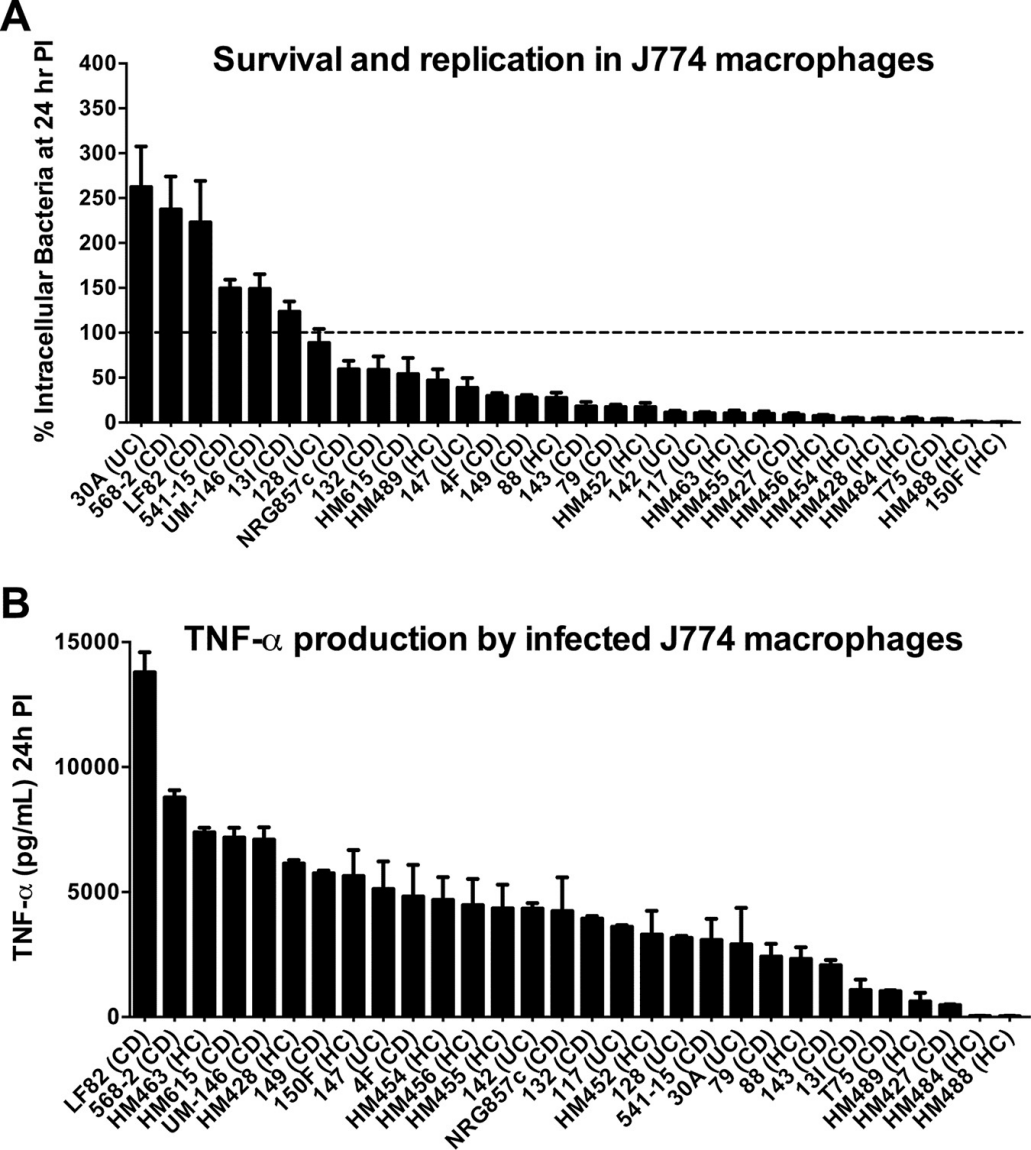
E. coli strains vary widely in their ability to survive and replicate in macrophages and to induce TNF-α production from infected macrophages *in vitro*. J774A.1 macrophages were infected with E. coli strains at an MOI of 10 for 2 h. (A) Survival and replication of E. coli strains. The percentage of intracellular bacteria at 24 h postinfection (PI) was calculated relative to that obtained at 1 h after gentamicin treatment (defined as 100% and shown as a dashed line). (B) TNF-α levels from infected J774 macrophages at 24 h postinfection. Data represent the mean of triplicate technical replicates ± SEM from at least three independent experiments.

*In vitro* survival and replication in the J774 macrophage cell line, production of TNF-α by infected J774 macrophages, and attachment, invasion, and replication in the Caco2 epithelial cell line were parameters first used by Darfeuille-Michaud and colleagues (10, 17, 27) to assess AIEC virulence traits. We systematically evaluated all 30 strains with a series of consistent *in vitro* phenotyping conditions based on the original methodology described by Darfeuille-Michaud (10, 17, 27). Assessment of strain survival and replication inside J774 macrophages at 24 h postinfection revealed that only 6 of the 30 strains were resistant to macrophage killing and possessed the capacity to replicate, with abundances at 24 h that were 262% to 123% relative to the number of intracellular bacteria recovered at 1 h after gentamicin treatment (defined as 100%) (Fig. 2A). The remaining 24 strains failed to replicate within macrophages and differed in their rates of survival from 88% to 0.6%.

As with intracellular survival and replication, we also observed a wide spectrum of TNF-α levels produced by E. coli-infected J774 macrophages at 24 h postinfection (Fig. 2B). Notably, there was no correlation between the amount of TNF-α produced and the survival and/or replication kinetics of the isolates inside J774 macrophages. For example, E. coli 13I survived and replicated in macrophages (123%) yet induced little TNF-α production from macrophages (1,083 pg/mL) compared to most other strains tested. Conversely, E. coli HM463 (7,387 pg/mL) induced over six times more TNF-α production than 13I but was readily killed by macrophages (10.4% survival).

**FIG 3:**
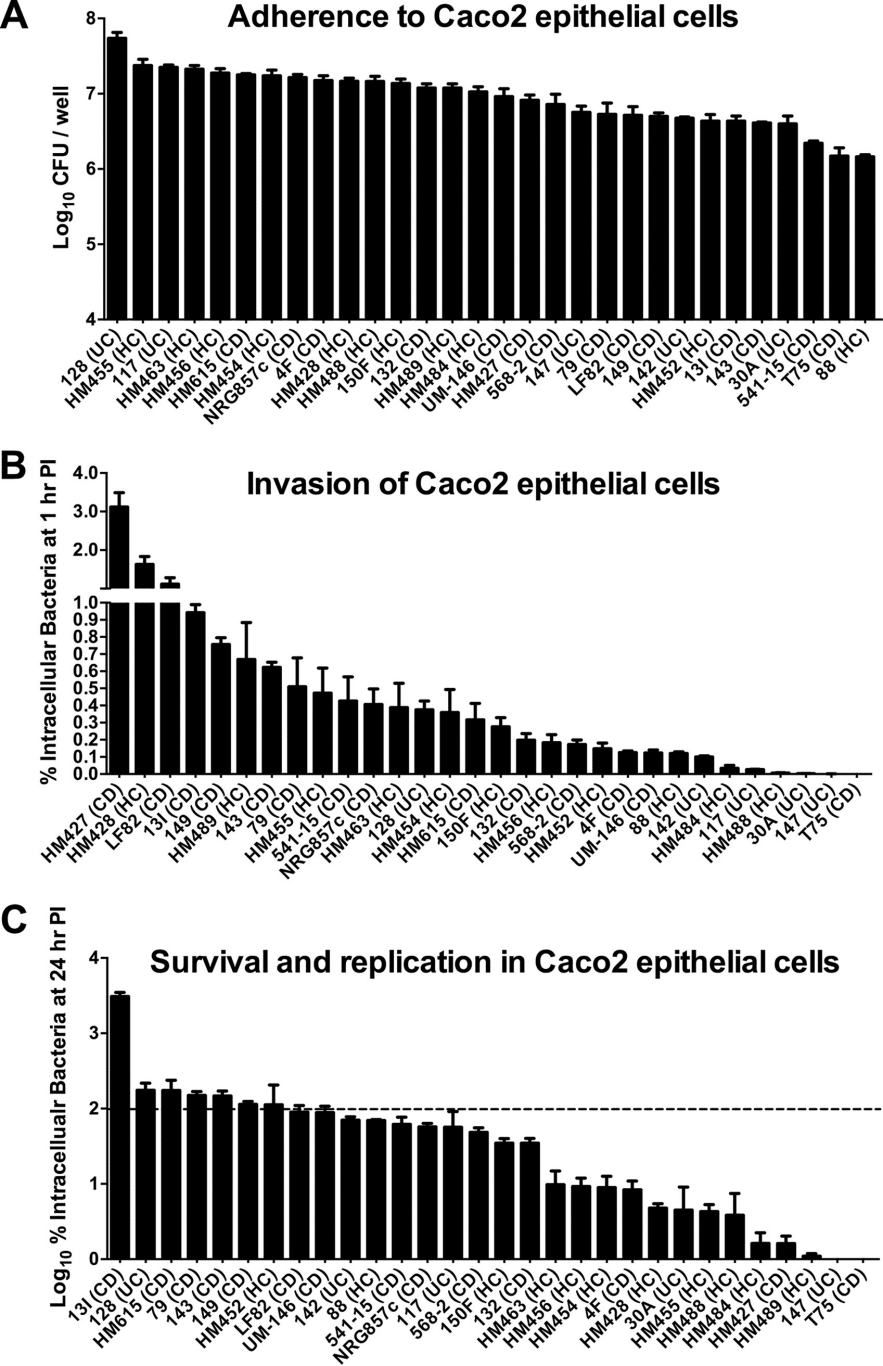
E. coli strains vary widely in their ability to invade and replicate in epithelial cells but not in adherence to epithelial cells. Caco2 cells were infected with E. coli strains at an MOI of 10 for 3 h. (A) Adherence of E. coli strains to the Caco2 monolayer. (B) Invasion of Caco2 cells by E. coli strains. The percentage of intracellular bacteria at 1 h after gentamicin treatment was calculated relative to that of the original inoculum. (C) Survival and replication of E. coli strains within Caco2 cells at 24 h postinfection. The percentage of intracellular bacteria at 24 h postinfection was calculated relative to that obtained at 1 h after gentamicin treatment (defined as 100% and shown as a dashed line). Values were log_10_ transformed. For all panels, data represent the mean of triplicate technical replicates ± SEM from at least three independent experiments.

In contrast to the highly variable phenotypes observed during macrophage assays, all 30 strains adhered to differentiated Caco2 epithelial cells, displaying only a 1.5-log range in adherence among strains (Fig. 3A). The majority of the strains tested (24 of 30) invaded Caco2 cells, with percentages of intracellular bacteria ranging from 3.1% to 0.1% of that of the original inoculum following a 1-h gentamicin treatment (Fig. 3B). Only six strains demonstrated little to no ability to invade (0.03% to 0%). All 24 strains that invaded Caco2 cells were capable of surviving inside those cells by 24 h postinfection (Fig. 3C). See Table S2 for detailed results from all *in vitro* screening assays.

10.1128/msphere.00478-22.5 TABLE S2 *In vitro* phenotypic screening of 30 mucosa-associated E. coli strains. Download Table S2, PDF file, 0.09 MB. Copyright © 2023 Kittana et al. 2023 Kittana et al. https://creativecommons.org/licenses/by/4.0/ This content is distributed under the terms of the Creative Commons Attribution 4.0 International license.

In summary, five of 30 strains (568-2, 541-15, UM-146, 13I, and LF82) replicated in J774 macrophages and invaded and replicated in Caco2 epithelial cells, thereby displaying a complete *in vitro* AIEC phenotype. An additional five strains (T75, 147, HM484, HM488, and 117) failed to replicate in macrophages and invade/replicate in epithelial cells and were therefore considered to have a clear non-AIEC *in vitro* phenotype. The remaining 20 strains were either able to invade epithelial cells but not replicate in macrophages (19 strains) or vice versa (1 strain, namely 30A), making a clear phenotypic differentiation of these 20 strains as either AIEC or non-AIEC impossible. Together, this phenotypic evaluation demonstrates a wide variation in AIEC phenotypes of E. coli isolates and an inability to clearly assign most isolates to an AIEC pathovar when screened using consistent parameters.

### E. coli strains exhibiting an AIEC phenotype *in vitro* exacerbated intestinal inflammation in mice while most non-AIEC strains did not.

To test the hypothesis that strains displaying an *in vitro* AIEC phenotype are more pathogenic than commensal E. coli strains and to determine the relevance of the different AIEC phenotypic traits associated with disease, we assessed strain virulence in a well-established murine model of intestinal inflammation inducible by E. coli (39–41). Gnotobiotic C3H/HeN mice harboring a defined microbiota (the altered Schaedler flora [ASF]) devoid of any *Enterobacteriaceae* can be colonized with E. coli for 3 weeks prior to exposure to 2.5% dextran sulfate sodium salt (DSS) for 5 days to trigger intestinal inflammation primarily in the cecum (40). We selected 18 of the intestinal mucosa-associated E. coli strains evaluated above, including five isolates displaying a complete *in vitro* AIEC phenotype, five displaying a non-AIEC phenotype, and several strains with an intermediate AIEC phenotype (i.e., either able to invade epithelial cells but not replicate in macrophages or vice versa). We also selected strains representing different E. coli clades. Each strain in this subset was then tested individually for the ability to exacerbate disease *in vivo*. Inflammation in mice was quantified by assigning macroscopic cecal scores based on gross observations of disease and cumulative histopathological cecal scores based on gland hyperplasia, stromal collapse, edema, cellular inflammation, ulceration, and mucosal height.

**FIG 4:**
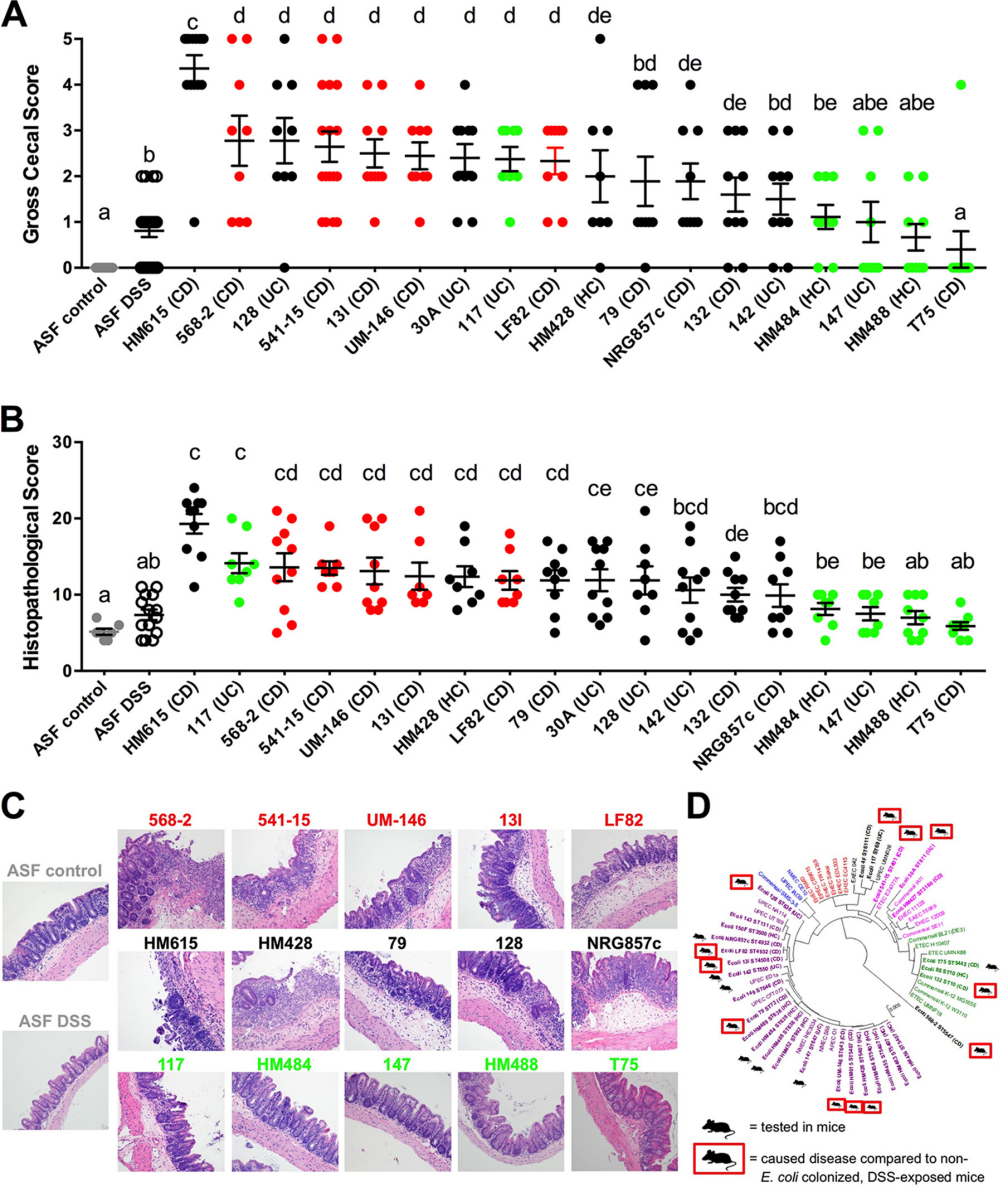
E. coli strains assigned an *in vitro* AIEC phenotype exacerbate intestinal inflammation in mice. (A) Macroscopic cecal lesion scores (*n* ≥ 10 mice per treatment). (B) Microscopic cecal lesion scores (*n* ≥ 5 mice per treatment). (C) Representative photomicrographs of hematoxylin-and-eosin-stained cecal tissues taken at ×20 objective magnification. (D) Diagram showing the distribution of disease-causing E. coli strains across different phylogroups. Data were analyzed using a nonparametric Kruskal-Wallis test followed by an unpaired Mann-Whitney *post hoc* test. Data represent the mean ± SEM. Red dots (A and B) and type (C) represent E. coli strains that replicated in J774 macrophages and invaded and replicated in Caco2 epithelial cells. Green dots (A and B) and type (C) represent strains that failed to replicate in macrophages and invade/replicate in epithelial cells. Black dots (A and B) and type (C) represent strains were either able to invade epithelial cells but not replicate in macrophages or vice versa. Treatments with unique letters (either a, b, c, d, or e) or combinations of letters are significantly different from one another at a *P* of <0.05.

As previously shown (40), DSS-treated ASF control mice not colonized with E. coli exhibited only mild intestinal inflammation compared to ASF control mice not receiving DSS (Fig. 4A to C). Comparison of ASF-bearing mice colonized with the different E. coli strains revealed differing susceptibilities to DSS-induced inflammation. The five E. coli strains that demonstrated an AIEC phenotype *in vitro* (568-2, 541-15, UM-146, 13I, and LF82) all significantly exacerbated intestinal inflammation in mice following DSS treatment compared to mice harboring no E. coli and exposed to DSS. In contrast, mice colonized individually with four of the five non-AIEC strains (T75, 147, HM484, or HM488) experienced only mild intestinal inflammation following DSS treatment that was similar in severity to that of non-E. coli-colonized control animals exposed to DSS. Only one strain with a non-AIEC *in vitro* phenotype, 117, caused severe lesions that were comparable to those caused by strains with an AIEC phenotype. Notably, E. coli strains that caused disease in mice did not cluster into a distinct phylogroup (Fig. 4D).

E. coli strains that exhibited only partial AIEC phenotypes *in vitro*, such as HM428, 79, 30A, 128, and 132, were capable of causing intestinal inflammation in mice similar to that observed for the five strains with a complete AIEC phenotype. Of those, strain HM615, which successfully invaded epithelial cells but did not replicate in macrophages, caused the most severe disease *in vivo* of any isolate tested.

Collectively, these results demonstrate that E. coli strains from a variety of distinct phylogenetic lineages can contribute to intestinal inflammation. Additionally, we observed that strains with an *in vitro* AIEC phenotype tended to induce greater levels of intestinal inflammation *in vivo* following DSS exposure than did non-AIEC strains. However, just as we observed for the *in vitro* phenotyping, there is no clear separation *in vivo* between the AIEC phenotype and the strains that possessed only some AIEC characteristics, as most of these intermediate isolates caused various levels of disease.

### Which *in vitro* phenotypic traits contribute to pathogenicity?

**FIG 5:**
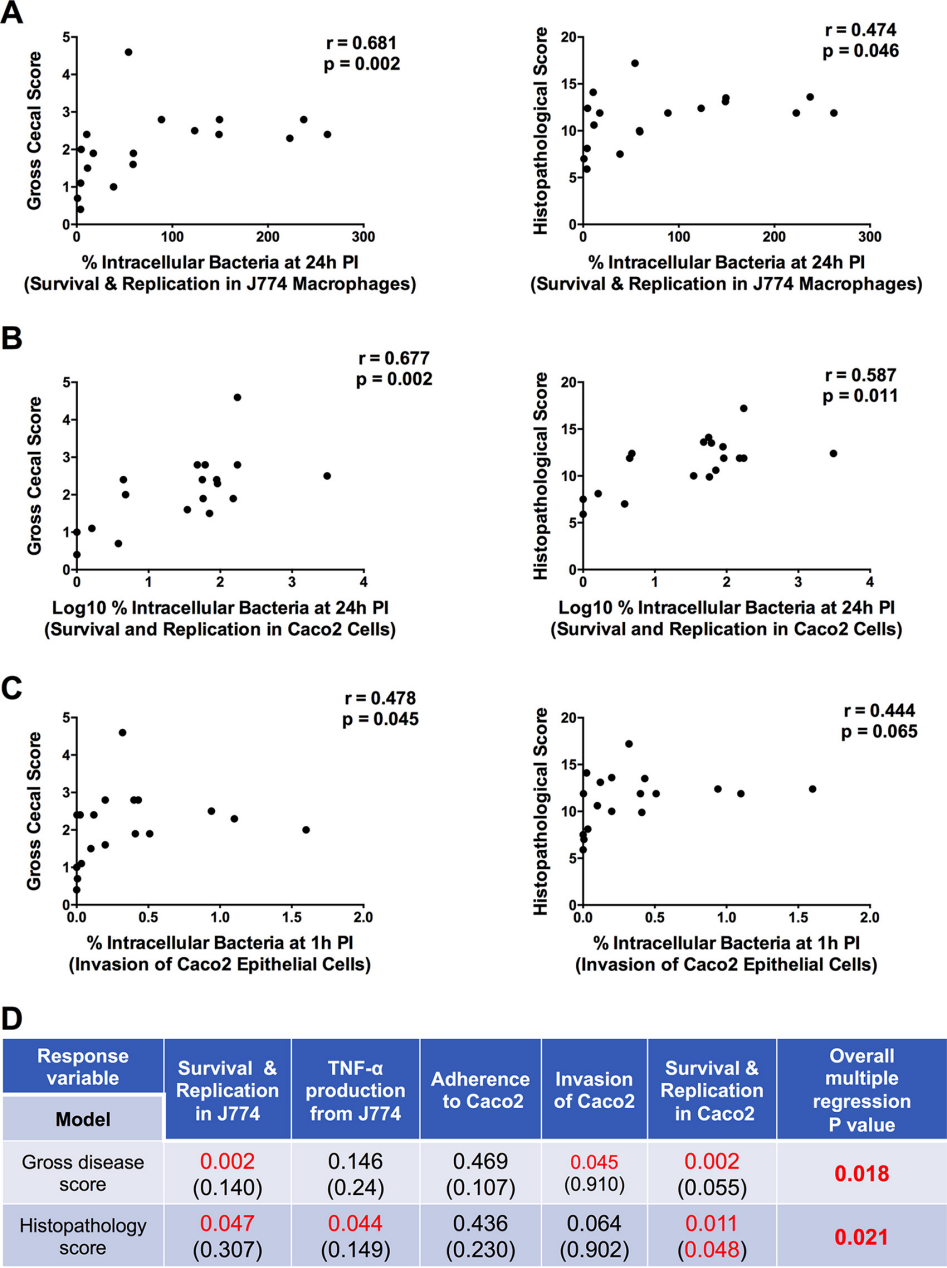
Associations between *in vitro* phenotypes and disease scores in a mouse model of intestinal inflammation. (A to C) Each dot represents the mean value for the two parameters evaluated (*in vivo* disease score on the *x* axis and *in vitro* phenotype on the *y* axis) using Spearman’s correlation analysis for each E. coli strain (*n* = 18). (D) Spearman’s correlation and multiple regression analyses. Each disease model (gross score and histopathological score) is shown in a single row, while each *in vitro* variable is represented by a single column. Numbers indicate *P* values for the significance of the Spearman correlation between each model and the response variable or the significance of each individual response variable within the multiple regression model (in parentheses). The rightmost column provides the *P* value for the overall multiple regression model of the *in vitro* response variables. Values below the significance level of *P < *0.05 are highlighted in red.

We next sought to determine the extent to which *in vitro* phenotypic characteristics correlated with the ability of E. coli strains to cause disease. Spearman’s correlation analysis revealed that replication in J774 macrophages was strongly positively correlated with macroscopic and microscopic lesions in mice (*r* = 0.68, *P = *0.002, and *r* = 0.47, *P = *0.047, respectively) (Fig. 5A). Production of TNF-α by E. coli-infected macrophages was positively correlated with microscopic scores (*r* = 0.48, *P = *0.044) but not with macroscopic disease scores (*r* = 0.36, *P = *0.146). No correlation was observed between adherence to Caco2 cells and gross or microscopic disease scores (*r* = 0.18, *P = *0.469, and *r* = 0.20, *P = *0.436, respectively), an observation consistent with the minimal differences in epithelial cell adherence levels seen among the strains. However, the ability of E. coli strains to invade epithelial cells was positively correlated with macroscopic cecal lesion development (*r* = 0.48, *P = *0.045) and tended to correlate with microscopic scores (*r* = 0.44, *P = *0.065) (Fig. 5B). Similar to replication in macrophages, replication of strains within epithelial cells was strongly positively correlated with severity of both macroscopic and microscopic lesions (*P = *0.002 and *P = *0.011, respectively) (Fig. 5C). An overall multiple regression analysis that included results from all five *in vitro* tests revealed that the *in vitro* phenotypes together could predict the disease-causing potential of strains in our animal model (*P = *0.018 and *P = *0.021 for macroscopic and microscopic disease scores, respectively) (Fig. 5D). Collectively, these results demonstrate a strong positive association between *in vitro* survival and replication of E. coli strains in J774 macrophages and Caco2 epithelial cells and their ability to cause disease in mice.

### Adherent non-AIEC strains protect against AIEC-mediated intestinal inflammation.

Previous studies have shown that various species in the gut microbiota can competitively exclude closely related strains through colonization resistance and/or niche exclusion (42, 43). Considering that many of the strains assessed in our assays had differential pathogenicity but similar levels of adherence to intestinal epithelial cells, we tested whether adherent, non-AIEC strains with low pathogenicity were capable of competing with a pathogenic strain for adherence *in vivo*, thereby preventing invasion and limiting disease severity.

**FIG 6:**
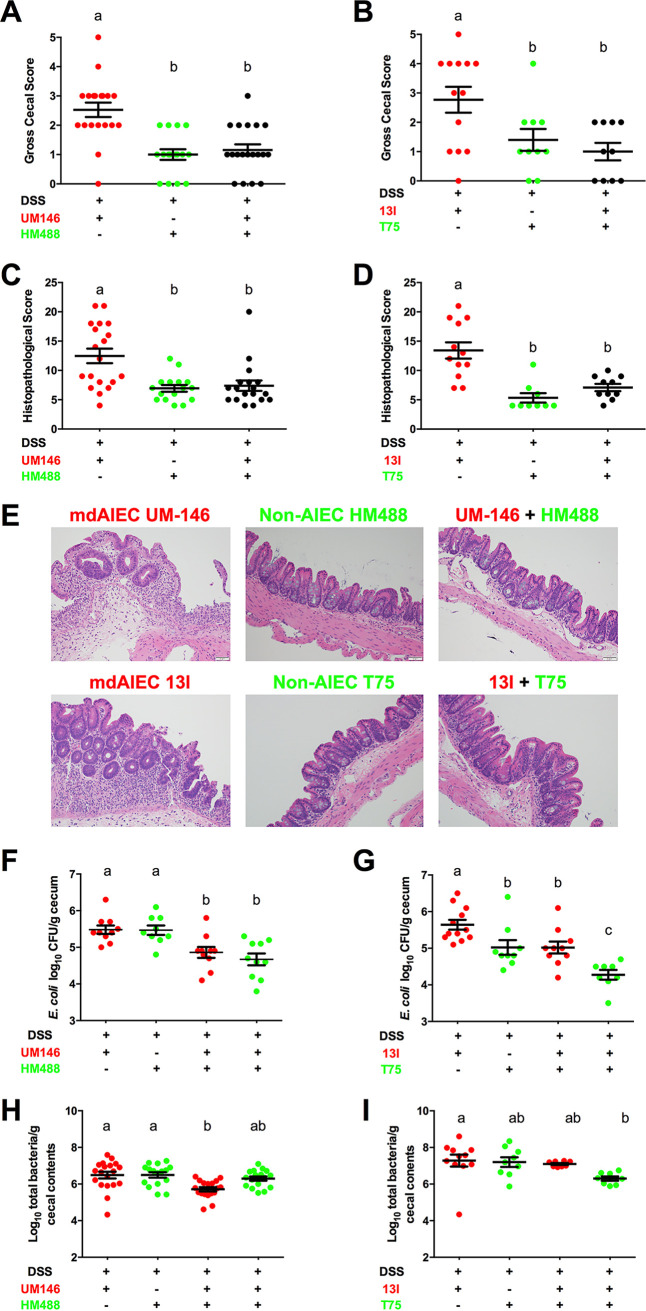
Cocolonization of non-AIEC strains with AIEC strains protected against AIEC-mediated intestinal inflammation. (A and B) Macroscopic cecal lesion scores of mice colonized with only an AIEC strain (UM146 or 13I), only a non-AIEC strain (HM488 or T75), or both and exposed to 2.5% DSS treatment (*n* ≥ 10 mice per treatment). (C and D) Microscopic cecal lesion scores (*n* ≥ 10 per treatment). (E) Representative photomicrographs of hematoxylin-and-eosin-stained cecal tissues were taken at a ×20 objective magnification. (F and G) E. coli adherence to cecal tissues (*n* ≥ 8 mice per treatment). Data in panels F and G were log_10_ transformed and are shown as the mean ± SEM. (H and I) Luminal abundance of E. coli in cecal contents as determined by quantitative PCR using strain-specific primers (*n* ≥ 8 mice per treatment). All data were analyzed using a nonparametric Kruskal-Wallis test followed by an unpaired Mann-Whitney *post hoc* test. Treatments with different lowercase letters are significantly different from one another at a *P* of <0.05.

We simultaneously colonized gnotobiotic ASF-bearing mice with strains displaying an AIEC and non-AIEC phenotype for 3 weeks prior to 2.5% DSS treatment. AIEC strains (13I and UM-146) and non-AIEC strains (T75 and HM488) were selected and paired together for the *in vivo* competition experiments based upon their complementary antibiotic susceptibility profiles. Consistent with the results shown in Fig. 4, mice colonized with AIEC phenotype strain 13I or UM-146 exhibited severe cecal lesions following DSS exposure whereas mice colonized with non-AIEC phenotype strain T75 or HM488 caused only minimal inflammation (Fig. 6A to E). Strikingly, cocolonization with the non-AIEC strain T75 and the AIEC strain 13I protected mice from severe disease, as evidenced by scores that were indistinguishable from those of mice colonized only with the non-AIEC strain. Similar results were observed when mice were cocolonized with HM488 (non-AIEC) and UM-146 (AIEC). A slight albeit significant reduction in adherence levels of AIEC strains 13I and UM-146 to cecal tissues was observed in mice cocolonized with both an AIEC strain and a non-AIEC strain compared to mice colonized only with either AIEC 13I or UM-146 (Fig. 6F and G). A similar pattern was observed when the luminal abundance of each E. coli was measured using strain-specific primers (Fig. 6H and I).

We next performed a series of *in vitro* studies to further examine the role of non-AIEC strains in limiting the adherence and abundance of AIEC strains. No differences in the growth of the AIEC strain 13I were observed when cultured in the absence or presence of the non-AIEC strain T75 (Fig. S1A). Similar results were also obtained for cocultures of UM-146 (AIEC) and HM488 (non-AIEC) (Fig. S1B). *In vitro* coinfection of Caco2 cells with AIEC 13I and non-AIEC T75 revealed no effect on the adherence and invasion patterns of 13I (AIEC) versus those of infections with 13I alone (Fig. S2A and B). The *in vitro* survival/replication of AIEC 13I in Caco2 and J774 cells was also unchanged when these cells were coinfected with T75 versus when they were infected alone (Fig. S2C and D). Although our *in vivo* findings suggest that adherent, non-AIEC strains have the potential to block the adherence of strains with an AIEC phenotype, our *in vitro* results suggest that more complex host-microbe interactions likely explain the adherent and luminal reductions in AIEC strain burden and protection against *in vivo* intestinal inflammation.

10.1128/msphere.00478-22.2 FIG S1 Coculturing AIEC and non-AIEC strains *in vitro* did not restrict growth of either strain. (A) Growth of AIEC UM146 and non-AIEC HM488 strains individually or together. (B) Growth of AIEC 13I and non-AIEC T75 strains individually or together. Data represent the mean of three technical replicates ± SEM of at least two independent experiments. Download FIG S1, PDF file, 0.2 MB. Copyright © 2023 Kittana et al. 2023 Kittana et al. https://creativecommons.org/licenses/by/4.0/ This content is distributed under the terms of the Creative Commons Attribution 4.0 International license.

10.1128/msphere.00478-22.3 FIG S2 *In vitro* coinfection of mammalian cells with AIEC and non-AIEC strains did not alter the AIEC phenotype. (A to C) Caco2 cells were infected with AIEC strain 13I, non-AIEC strain T75, or both strains at an MOI of 5 for each strain for 3 h. (A) Adherence of E. coli strains to the Caco-2 monolayer. (B) Invasion of Caco2 cells by E. coli strains. The percentage of intracellular bacteria at 1 h after gentamicin treatment was calculated relative to that of the original inoculum. PI, postinfection. (C) Survival and replication of E. coli strains within Caco2 cells at 24 h postinfection. The percentage of intracellular bacteria at 24 h postinfection was calculated relative to that obtained at 1 h after gentamicin treatment (defined as 100% and shown as a dashed line). (D) J774 murine macrophage cells were infected with AIEC strain 13I, non-AIEC strain T75, or both strains at an MOI of 5 for each strain for 2 h. Survival and replication of E. coli strains are shown. The percentage of intracellular bacteria at 24 h postinfection was calculated relative to that obtained at 1 h after gentamicin treatment (defined as 100%). Data represent the mean of five technical replicates ± SEM from at least four independent experiments. Download FIG S2, PDF file, 0.3 MB. Copyright © 2023 Kittana et al. 2023 Kittana et al. https://creativecommons.org/licenses/by/4.0/ This content is distributed under the terms of the Creative Commons Attribution 4.0 International license.

## DISCUSSION

In this study, we utilized a combination of *in vitro* phenotyping and a murine model of intestinal inflammation to systematically compare E. coli strains identified as AIEC with those that were not and then related the *in vitro* phenotypes used to make this differentiation to pathogenicity. We found that E. coli strains displaying *in vitro* AIEC phenotypes caused, on average, more severe intestinal inflammation. Additionally, survival and replication of strains in J774 macrophages and Caco2 epithelial cells were positively correlated with disease *in vivo*, while adherence to Caco2 cells and TNF-α production by J774 cells were not. Our results support the hypothesis that AIEC strains are more pathogenic than commensal E. coli strains and implicate the importance of intracellular survival and replication in their pathogenicity. However, we did not detect a clear phylogenetic or functional separation between AIEC isolates and commensal E. coli—an observation consistent with previous studies that describe a high degree of variability in AIEC phenotypes and putative virulence genes among strains (9, 13, 15).

This continuum of phenotypes agrees with the evolution of E. coli as a species. E. coli utilizes highly complex and dynamic adaptive strategies in which genetic material is constantly exchanged among different lineages to generate a larger pangenome (44). Genes that prove beneficial under certain ecological conditions are therefore likely selected and maintained, but the selective forces are possibly too dynamic for divergent lineages to evolve distinct and coherent genetic and phenotypic adaptations (45). These evolutionary insights are consistent with our findings and those of others showing that AIEC isolates are paraphyletic and that no specific virulence genes or unique molecular determinants have been identified for this pathotype (13, 15). These insights also explain why AIEC strains identified to date do not form a pathovar that is clearly separated from commensal E. coli and why these strains cannot yet be identified by core genes, molecular properties, or phenotypes. Instead, dynamic evolutionary paths have created strains that are genetically, phylogenetically, and functionally variable and possess different gene sets (44, 46, 47). Some of these genes appear to allow for enhanced fitness across phylogenetic backgrounds under inflammatory conditions in the gut and likely encode the AIEC phenotypes that we and others have studied (48–50). Together, these ideas and observations strongly suggest that the expansion of E. coli strains with AIEC characteristics likely results from an inflammatory environment.

In this study, we demonstrate that the importance of AIEC phenotypes goes beyond an ecological role that promotes dominance during inflammation but that it also contributes to disease pathogenesis. Although assigning causality in microbiome studies is challenging (51–54), we showed that an appropriate animal model can be used to test the hypothesis that AIEC strains have an enhanced ability to cause disease compared to other gut commensal E. coli. The five strains we identified as exhibiting a clear AIEC phenotype as well as six intermediate strains (five that replicated in macrophages but not epithelial cells and one that replicated in epithelial cells but not macrophages) exacerbated intestinal inflammation in gnotobiotic mice exposed to DSS compared to control mice treated with DSS, while only one of the five non-AIEC strains identified in our screen enhanced intestinal inflammation in an animal model. Our methodology represents a variation of Koch’s postulates where a candidate, disease-promoting, commensal strain isolated from a human with a specific disease was introduced into a new host to determine if it exacerbated disease (51, 53). The systematic application of this approach to a collection of strains with a spectrum of *in vitro* phenotypes demonstrates that commensal strains with an AIEC phenotype have a greater propensity to cause disease than commensal E. coli strains found in the same ecological microhabitat. Moreover, this approach also helped identify bacterial traits likely to contribute to AIEC virulence. Although our current work does not specifically assign disease causality for AIEC in humans, it does fulfill the majority of Koch’s postulates and thus provides strong evidence for a causal link between E. coli intracellular survival/replication and pathogenicity in a mouse model.

Despite the substantial variability observed among strains in the *in vitro* phenotypes previously used to identify AIEC, our data do indicate that some of these phenotypes are still pathologically relevant. We found strong associations between E. coli survival and replication in macrophages and epithelial cells *in vitro* and strain pathogenicity *in vivo*. These associations can potentially be explained by the initiation of inflammatory cascades aimed at restricting and eliminating bacterial invaders taken up by macrophages and other mammalian cells. Such pathways may not be effective in some IBD patients, as macrophages isolated from CD patients have been shown to be defective in bacterial clearance (55, 56). Therefore, the inflammatory responses that ensue following innate immune detection of intracellular bacteria may be aberrant in IBD patients, subsequently supporting bacterial replication and exacerbating disease that would not be possible in healthy individuals (8, 35). The high variability of AIEC phenotypes among E. coli strains, especially those isolates contributing to inflammation, may result from the complex ecological forces that shape E. coli evolution and the dynamic nature of the selective pressures present in the gut. E. coli lineages are not stably maintained within hosts—they switch hosts and even host species regularly (38). Consequently, E. coli is not consistently exposed, over evolutionary timescales, to the inflammatory conditions that locally and temporally select for AIEC phenotypes (49), not even in IBD patients who experience episodes of remission and relapse. In contrast, the adherence phenotype, which was not associated with disease, showed remarkably little variability among strains, possibly because all the strains in our study were isolated from mucosal biopsy specimens. The prevalence of this trait could represent selection for a specific niche characteristic that requires adherence to epithelial cells.

By understanding the relevance of AIEC phenotypes to pathogenicity, we were able to design a rational preventive strategy for AIEC-mediated inflammation in mice by capitalizing on our observation that epithelial adhesion was not associated with disease. By colonizing mice with an adherent, non-AIEC strain with low pathogenicity, we were able to limit the severity of AIEC-mediated disease. Our *in vivo* results further suggest that the non-AIEC strains were protective, in part, because they were capable of competing with an AIEC strain for adherence to the intestinal epithelium. Direct competition for colonization and adherence sites (niche displacement), direct killing via secretion of antimicrobial substances, and competition for nutrient sources are among the common strategies utilized by bacterial strains to competitively exclude similar or closely related strains (57). Our findings and those of others suggest that AIEC strains may be targeted by approaches that include administration of adherent, non-disease-causing E. coli strains or compounds that limit bacterial adhesion to the epithelium (58, 59). However, our *in vitro* coinfection results highlight the importance of considering that adherent non-AIEC strains may prevent disease via mechanisms independent of competition with AIEC for adherence sites. Kim and colleagues showed that microbial adhesion of E. coli promoted interleukin-10 (IL-10) production and limited proinflammatory T helper 1 cell responses (60). It is therefore possible that adherent non-AIEC limits AIEC-mediated inflammation and sustains intestinal homeostasis by inducing immunoregulatory phenotypes.

Although it is difficult to experimentally determine if the enrichment of AIEC strains during inflammation in human IBD is a cause and not simply a consequence of disease, our work now demonstrates that the phenotypes enriched in strains isolated from human patients do contribute to pathogenicity in a preclinical model of colitis. Our findings therefore suggest that these strains may also contribute to IBD pathogenesis in humans. As we demonstrate, information regarding the importance of *in vitro* AIEC phenotypes to strain pathogenicity can be used to develop therapeutic strategies. The ability to differentiate AIEC phenotypes that are pathologically relevant from those that are not provides an important foundation for the development of strategies to predict, diagnose, and treat human IBD by characterizing and modulating patient E. coli populations. However, more research is needed to determine the molecular signatures responsible for the phenotypes we have identified to be important for pathogenicity.

## MATERIALS AND METHODS

### Bacterial strains.

All E. coli strains used in this study were isolated from intestinal biopsy specimens of either CD or UC patients or from healthy control (HC) subjects (14 CD, 5 UC, and 11 HC) during previous investigations (see Table S1 in the supplemental material). See Text S1 in the supplemental material for additional details on culturing E. coli to inoculate mice.

10.1128/msphere.00478-22.1 TEXT S1 Supplemental methods. Download Text S1, PDF file, 0.2 MB. Copyright © 2023 Kittana et al. 2023 Kittana et al. https://creativecommons.org/licenses/by/4.0/ This content is distributed under the terms of the Creative Commons Attribution 4.0 International license.

### Cell culture assays.

The J774A.1 macrophage cell line (ATCC TIB-6) was used to assess the ability of E. coli strains to survive and replicate within macrophages and to induce TNF-α production from infected macrophages. The Caco2 intestinal epithelial cell line (ATCC HTB-37) was used to assess E. coli adherence to, invasion of, and survival in intestinal epithelial cells. We followed the methods described by Boudeau et al. (17), Glasser et al. (27), and Darfeuille-Michaud et al. (10) for phenotypic characterization of E. coli strains. Briefly, J774 cells were infected with E. coli strains at a multiplicity of infection (MOI) of 10 for 2 h, washed, and then provided with fresh medium supplemented with 100 μg/mL gentamicin for 1 h to kill extracellular bacteria. Levels of intracellular bacteria were assessed at 1 and 24 h postinfection. For cultures incubated for 24 h postinfection, medium containing 100 μg/mL gentamicin was removed at 1 h postinfection and replaced with medium containing 20 μg/mL gentamicin. At each time point, gentamicin-containing medium was removed, and the macrophages were lysed by adding 1 mL of 1% Triton X-100 in phosphate-buffered saline (PBS) to each well. Lysates were plated on eosin-methylene blue (EMB) agar to enumerate colonies. The percentage of intracellular bacteria at 24 h was calculated relative to that at 1 h after gentamicin treatment (defined as 100%). A protocol similar to that used for the J774 infection assays was also applied to the Caco2 infection assays, except that the infection period was 3 h instead of 2 h. Also, no gentamicin treatment was applied to cultures of Caco2 cells used to evaluate E. coli adherence. See the supplemental material for additional details.

### Mice.

Male and female C3H/HeN mice (8 to 10 weeks old) harboring the altered Schaedler flora microbiota from birth were bred and maintained under gnotobiotic conditions in the Nebraska Gnotobiotic Mouse Program as previously described (61). Three weeks after E. coli colonization, intestinal inflammation was triggered in mice by administering 2.5% dextran sulfate sodium salt (DSS; MP Biomedicals, OH) via their drinking water as previously described (40). Mice received 2.5% DSS for 5 consecutive days and then regular drinking water for 4 days prior to necropsy. All procedures involving animals were approved by the Institutional Animal Care and Use Committee at the University of Nebraska-Lincoln (protocol no. 817, 1215, and 1700).

### Gross and histopathological disease scores in mice.

To assess disease in mice following E. coli colonization, cecal tissues were harvested at necropsy and assigned a gross disease score in accordance with the parameters described by Gomes-Neto et al. (61), including atrophy, emptying, enlargement of the cecal tonsil, presence of mucoid contents, and presence of intraluminal blood. To assess microscopic lesions, the apical portions of cecal tissues were collected, fixed in 10% neutral buffered formalin (Thermo Fisher Scientific), and subsequently processed, sectioned, and stained with hematoxylin and eosin. Tissues were scored by a board-certified veterinary pathologist (J. M. Hostetter), who was blinded to the treatments. Cumulative histopathological scores ranged from 0 to 30 and were based on a score of 0 to 5 for each of the following parameters as previously described: gland hyperplasia, stromal collapse, edema, cellular inflammation, ulceration, and mucosal height (61). Higher cumulative scores represented more severe disease.

### Mouse cocolonization experiments to establish therapeutic potential of E. coli strains.

E. coli strains used for *in vivo* cocolonization studies (AIEC strains 13I and UM-146 and non-AIEC strains T75 and HM488) were selected and paired based upon their natural antibiotic susceptibility profiles. Specifically, AIEC strain 13I is resistant to oxytetracycline, while non-AIEC strain T75 is sensitive. AIEC strain UM-146 is sensitive to ampicillin, whereas non-AIEC HM488 is resistant (additional details are available in Text S1 in the supplemental material).

ASF-bearing C3H/HeN mice were colonized with either an AIEC strain alone or a non-AIEC strain alone or were cocolonized with both an AIEC strain and a non-AIEC strain via oral gavage as described in Text S1 in the supplemental material. Three weeks later, intestinal inflammation was triggered by DSS treatment as described above. Methods for determining E. coli adherence to cecal tissue and quantifying luminal levels are described in the supplemental material (Text S1).

### Statistical analysis.

All data are presented as the mean ± standard error of the mean (SEM). A nonparametric Kruskal-Wallis test followed by an unpaired Mann-Whitney *post hoc* test was used to analyze all data sets. Correlations between *in vitro* phenotypic assays and all disease scores were calculated using Spearman’s rank correlation and by individual and multiple regression analyses. All statistical analyses were performed using GraphPad Prism 6 (version 6.01; GraphPad, CA, USA) except for the regression analyses, which were performed using R software (version 3.4.1; R Foundation for Statistical Computing, Vienna, Austria). Differences were considered significant at a *P* of <0.05.
