# Supplementary material for: Evidence for a Causal Role for Escherichia coli Strains Identified as Adherent-Invasive (AIEC) in Intestinal Inflammation
Source: mSphere. 2023 Mar 8;8(2):e00478-22. doi: 10.1128/msphere.00478-22 (PMC10117065; doi:10.1128/msphere.00478-22)
Supplement: TABLE S1 [file msphere.00478-22-s0004.pdf]

Supplemental Table 1 Mucosa-associated *E. coli* strains used in this study

E. coli strain	Origin*	Phylogroup	Sequence Type**	Publication	Published Phenotype***
**541-15**	CD	A, B1	401	(1)	AIEC
**568-2**	CD	D	5547	(1)	AIEC
**T75**	CD	A	5442	(1)	Non-AIEC
**LF82**	CD	B2	4932	(2)	
**79**	CD	B2	73	(3)	
**88**	HC	A	10	(3)	
**117**	UC	D	69	(3)	
**128**	UC	B2	636	(3)	
**132**	CD	A	10	(3)	
**142**	UC	B2	550	(3)	
**143**	CD	B2	131	(3)	
**147**	UC	B2	647	(3)	
**149**	CD	B2	646	(3)	
**UM-146**	CD	B2	643	(4)	AIEC
**HM427**	CD	B1	3160	(5)	
**HM428**	HC	B2	5407	(5)	
**HM452**	HC	B2	992	(5)	
**HM454**	HC	B2	5407	(5)	
**HM455**	HC	B2	5407	(5)	
**HM456**	HC	B2	5407	(5)	
**HM463**	HC	B2	5407	(5)	
**HM484**	HC	B2	638	(5)	
**HM488**	HC	B2	638	(5)	
**HM489**	HC	B2	638	(5)	
**HM615**	CD	B2	5407	(5)	AIEC
**4F**	CD	D	6111	(6)	
**13I**	CD	B2	4508	(6)	
**30A**	UC	B1	871	(6)	
**150F**	HC	B2	3500	(6)	
**NRG857c**	CD	B2	4932	(7)	AIEC

*CD = Crohn's disease patient, UC=Ulcerative colitis patient, HC= healthy control individual

**Sequence type was generated based on the multilocus sequence type (MLST) scheme of Wirth *et al* (8)

****E. coli* strains previously screened and reported as either adherent-invasive *E. coli* (AIEC) or non-AIEC

Supplemental Table 1 References

1. Dogan B, Suzuki H, Herlekar D, Sartor RB, Campbell BJ, Roberts CL, Stewart K, Scherl EJ, Araz Y, Bitar PP, Lefebure T, Chandler B, Schukken YH, Stanhope MJ, Simpson KW. 2014. Inflammation-associated adherent-invasive *Escherichia coli* are enriched in pathways for use of propanediol and iron and M-cell translocation. Inflamm Bowel Dis 20:1919-32.
2. Glasser AL, Boudeau J, Barnich N, Perruchot MH, Colombel JF, Darfeuille-Michaud A. 2001. Adherent invasive *Escherichia coli* strains from patients with Crohn's disease survive and replicate within macrophages without inducing host cell death. Infect Immun 69:5529-37.
3. Sepehri S, Kotlowski R, Bernstein CN, Krause DO. 2009. Phylogenetic analysis of inflammatory bowel disease associated *Escherichia coli* and the fimH virulence determinant. Inflamm Bowel Dis 15:1737-45.
4. Kotlowski R, Bernstein CN, Sepehri S, Krause DO. 2007. High prevalence of *Escherichia coli* belonging to the B2+D phylogenetic group in inflammatory bowel disease. Gut 56:669-75.
5. Subramanian S, Rhodes JM, Hart CA, Tam B, Roberts CL, Smith SL, Corkill JE, Winstanley C, Virji M, Campbell BJ. 2008. Characterization of epithelial IL-8 response to inflammatory bowel disease mucosal *E. coli* and its inhibition by mesalamine. Inflamm Bowel Dis 14:162-75.
6. Sasaki M, Sitaraman SV, Babbin BA, Gerner-Smidt P, Ribot EM, Garrett N, Alpern JA, Akyildiz A, Theiss AL, Nusrat A, Klapproth JM. 2007. Invasive *Escherichia coli* are a feature of Crohn's disease. Lab Invest 87:1042-54.
7. Eaves-Pyles T, Allen CA, Taormina J, Swidsinski A, Tutt CB, Jezek GE, Islas-Islas M, Torres AG. 2008. *Escherichia coli* isolated from a Crohn's disease patient adheres, invades, and induces inflammatory responses in polarized intestinal epithelial cells. Int J Med Microbiol 298:397-409.
8. Wirth T, Falush D, Lan R, Colles F, Mensa P, Wieler LH, Karch H, Reeves PR, Maiden MC, Ochman H, Achtman M. 2006. Sex and virulence in *Escherichia coli*: an evolutionary perspective. Mol Microbiol 60:1136-51.
