# Supplementary material for: Evidence for a Causal Role for Escherichia coli Strains Identified as Adherent-Invasive (AIEC) in Intestinal Inflammation
Source: mSphere. 2023 Mar 8;8(2):e00478-22. doi: 10.1128/msphere.00478-22 (PMC10117065; doi:10.1128/msphere.00478-22)
Supplement: TABLE S2 [file msphere.00478-22-s0005.pdf]

Supplemental Table 2 *In vitro* phenotypic screening of 30 mucosa-associated *E. coli* strains (values represent triplicate technical replicates from at least 3 independent experiments).

***E. coli* strain**	**Replication in J774 (%)**	**TNF-α Production by J774 (pg/mL)**	**Adherence to Caco2 (log_{10})**	**Replication in Caco2 (log_{10}%)**	**Invasion in Caco2 (%)**
**541-15**	149.38	3087	6.34	1.79	0.43
**568-2**	237.42	8781	6.86	1.68	0.17
**T75**	3.97	1033	6.17	0.00	0.00
**LF82**	223.00	13791	6.71	1.96	1.12
**79**	17.30	2415	6.73	2.18	0.51
**88**	27.30	2331	6.16	1.84	0.12
**117**	10.64	3610	7.35	1.75	0.03
**128**	88.67	3168	7.74	2.24	0.38
**132**	59.00	3942	7.08	1.54	0.20
**142**	11.40	4341	6.68	1.85	0.10
**143**	18.01	2073	6.61	2.17	0.62
**147**	38.64	5122	6.75	0.00	0.00
**149**	27.94	5755	6.70	2.06	0.76
**UM-146**	148.93	7095	6.96	1.95	0.12
**HM427**	8.57	476	6.91	0.21	3.12
**HM428**	4.66	6143	7.16	0.68	1.63
**HM452**	17.30	3305	6.64	2.05	0.15
**HM454**	4.94	4689	7.24	0.95	0.36
**HM455**	9.84	4352	7.37	0.95	0.47
**HM456**	7.25	4475	7.28	0.63	0.18
**HM463**	10.39	7387	7.33	0.99	0.39
**HM484**	4.18	44	7.03	0.21	0.03
**HM488**	0.87	34	7.16	0.58	0.01
**HM489**	46.97	623	7.08	0.04	0.67
**HM615**	54.15	7182	7.25	2.24	0.32
**4F**	29.83	4821	7.18	0.92	0.13
**13I**	123.42	1083	6.63	3.49	0.94
**30A**	262.34	2914	6.60	0.65	0.00
**150F**	0.64	5643	7.14	1.54	0.28
**NRG857c**	59.31	4240	7.21	1.76	0.41
