# Supplementary figures and images for: Evidence for a Causal Role for Escherichia coli Strains Identified as Adherent-Invasive (AIEC) in Intestinal Inflammation
Source: mSphere. 2023 Mar 8;8(2):e00478-22. doi: 10.1128/msphere.00478-22 (PMC10117065; doi:10.1128/msphere.00478-22)

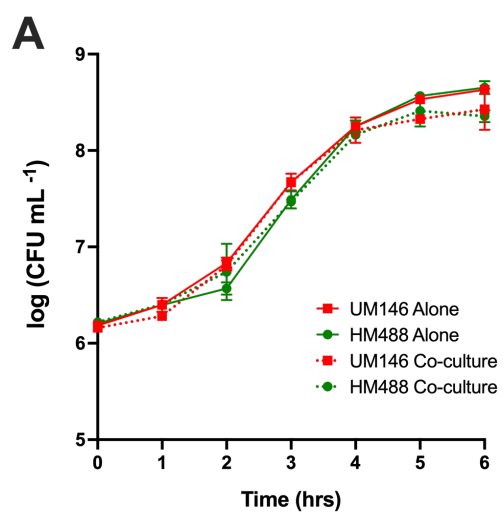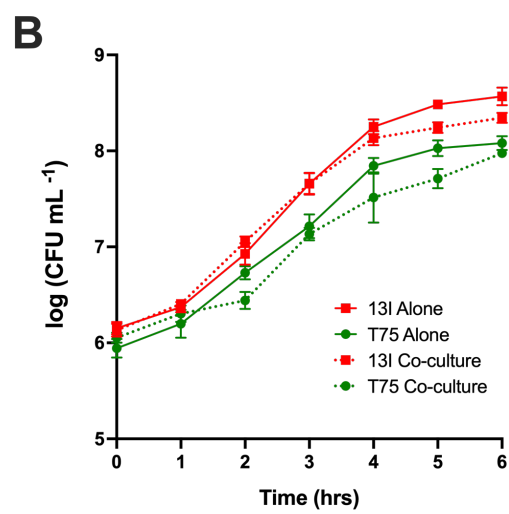

Supplement: FIG S1 [file msphere.00478-22-s0002.pdf]

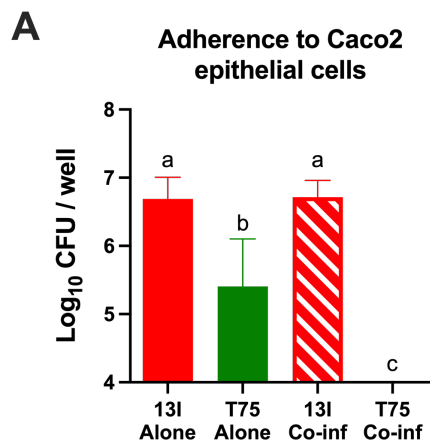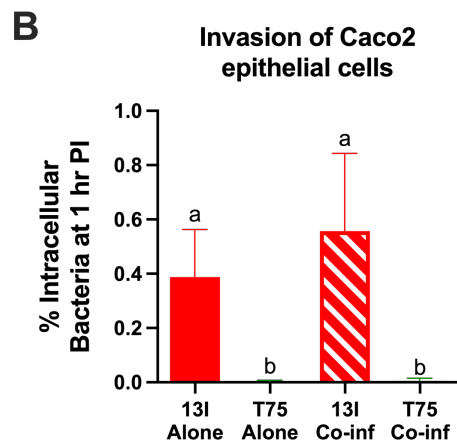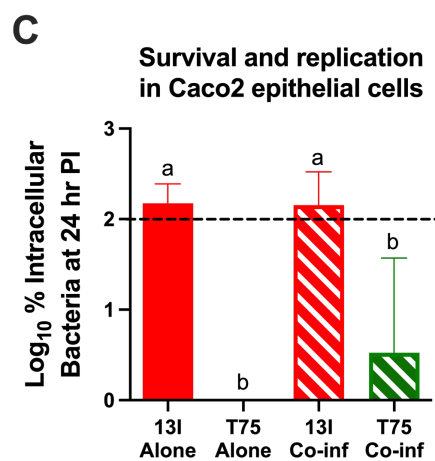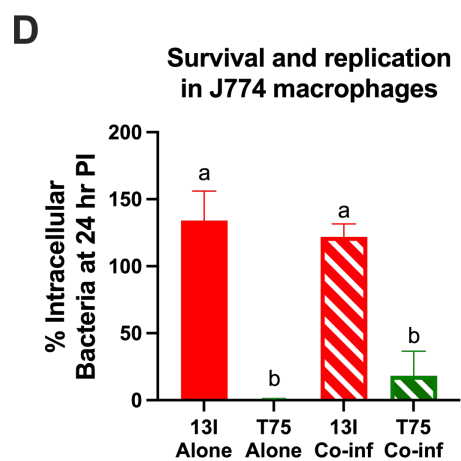

Supplement: FIG S2 [file msphere.00478-22-s0003.pdf]
