# Supplementary material for: Evidence for a Causal Role for Escherichia coli Strains Identified as Adherent-Invasive (AIEC) in Intestinal Inflammation
Source: mSphere. 2023 Mar 8;8(2):e00478-22. doi: 10.1128/msphere.00478-22 (PMC10117065; doi:10.1128/msphere.00478-22)
Supplement: TEXT S1 [file msphere.00478-22-s0001.pdf]

Supplemental Methods

Evidence for a causal role for *Escherichia coli* strains identified as adherent-invasive (AIEC) in intestinal inflammation

Hatem Kittana, João C. Gomes-Neto, Kari Heck, Anthony F. Juritsch, Jason Sughroue, Yibo Xian, Sara Mantz, Rafael R. Segura Muñoz, Liz A. Cody, Robert J. Schmaltz, Christopher L. Anderson, Rodney A. Moxley, Jesse M. Hostetter, Samodha C. Fernando, Jennifer Clarke, Stephen D. Kachman, Clayton E. Cressler, Andrew K. Benson, Jens Walter and Amanda E. Ramer-Tait

Bacterial strains and mice

For mouse experiments, *E. coli* strains were grown overnight at 37°C on EMB agar plates (Difco, NJ, USA). The following day, a colony was selected to inoculate a 10 mL sterile Luria Bertani (LB) broth that was incubated overnight at 37°C. One mL of each overnight culture was transferred to a fresh 10 mL LB broth culture and incubated at 37°C for 4 to 6 hr (late log phase) with shaking at 200 rpm. Bacterial cultures were then centrifuged and resuspended at an approximate concentration of 1×10^8 colony forming unit (CFU) in 200 µL of LB broth for oral gavage.

The male and female C3H/HeN mice used in this study harbored the Altered Schaedler Flora (ASF) community, which included: ASF 356, *Clostridium sp.*; ASF 360, *Lactobacillus intestinalis*; ASF361, *Lactobacillus murinus*; ASF 457, *Mucispirillum schaedleri*; ASF 492, *Eubacterium plexicaudatum*; ASF 500, *Pseudoflavonifractor* sp.; ASF 502, *Clostridium* sp.; and ASF 519, *Parabacteroides goldsteinii* (1, 2). Prior to inoculation with *E. coli*, ASF-bearing mice were transferred from flexible film isolators to a positive pressure, individually-ventilated caging system and maintained as previously reported (3). Each mouse received 1×10^8 *E. coli* CFU in 200 µL of LB broth via oral gavage. Successful *E. coli* colonization was verified by collecting fecal samples 10 days post-inoculation and plating on Eosin Methylene Blue (EMB) agar plates (Difco). All control ASF-bearing mice were confirmed to be *E. coli* free by plating fecal samples.

DNA extraction, multilocus sequence typing (MLST) and phylogenetic analysis

Genomic DNA was extracted from all *E. coli* strains using a QIAamp DNA Blood and Tissue Kit (Qiagen, MD, USA) as per manufacturer instructions. MLST analysis was performed using nucleotide sequences from the seven housekeeping genes (*adk*, *fumC*, *gyrB*, *icd*, *mdh*, *purA*, *recA*) according to the Achtman scheme (4) to determine allelic numbers and sequence types (ST). MLST-PCR products were purified using QIAquick PCR purification kit (Qiagen) and sequenced by Eurofins Genomics (Louisville, KY, USA). Sequences were trimmed, aligned and concatenated to give 3,423-nucleotide-long sequences using the BioEdit program (5). The construction of a maximum likelihood (ML) phylogeny tree utilizing concatenated MLST sequences was conducted based on the Tamura-Nei model (6) using Molecular Evolutionary Genetics Analysis (MEGA) software (7). MLST sequences of a total of 30 *E. coli* strains representing the 6 different *E. coli* phylogroups (A, B1, B2, D, E, and F) were obtained from the enterobase *E. coli* MLST database at University of Warwick (http://enterobase.warwick.ac.uk) and were incorporated in the ML tree to determine the phylogroups of *E. coli* strains used in the study. The selection of reference *E. coli* strains was determined based upon organism origin, where only *E. coli* originally isolated from human samples were included in the phylogeny tree.

Cell culture assays

The J774A.1 macrophage cell line (ATCC TIB-6) was obtained from the American Type Culture Collection (Manassas, VA) and used to assess the ability of *E. coli* strains to survive and replicate within macrophages as well as induce TNF-α production from infected macrophages. The Caco2 intestinal epithelial cell line (ATCC HTB-37) was also obtained from ATCC and used to assess *E. coli* adherence to, invasion of and survival in intestinal epithelial cells. We followed the methods described by Boudeau *et al*. (8), Glasser *et al.* (9), and Darfeuille-Michaud *et al.* (10) for

phenotypic characterization of *E. coli* strains. J774 macrophages were seeded at a density of 2 x 10^5 cells per well of a 24-well tissue culture plate in 500 µL of complete tissue culture medium (CTCM) containing Dulbecco's modified Eagle's medium containing 4.5 mg of glucose/mL, 2 mM L-glutamine, 100 U penicillin, 100 g streptomycin/mL, 25 mM HEPES, 0.05 M 2-mercaptoethanol and 10% fetal bovine serum. J774 cells were infected with *E. coli* strains at a MOI of 10 for 2 hr at 37°C and 5% CO_2. After 2 hr, J774 cells were washed twice with 1X PBS and provided fresh CTCM media supplemented with 100 µg/mL gentamicin for 1 hr to kill extracellular bacteria. Levels of intracellular bacteria were assessed at 1 and 24 hr post-infection. For cultures incubated for 24 hr post-infection, the CTCM containing 100 µg/mL gentamicin was removed at 1 hr post-infection and replaced with CTCM containing 20 µg/mL gentamicin. At each time point, gentamicin-containing media was removed, and the macrophages lysed by adding 1 mL of 1% Triton-X-100 in PBS to each well. Ten-fold serial dilutions of cell lysates were plated in 10 µL volumes in triplicate on EMB agar plates and incubated overnight at 37°C prior to enumerating colonies. The percentage of intracellular bacteria at 24 hr was calculated relative to 1 hr post-gentamicin treatment (defined as 100%). A higher gentamicin concentration (300 µg/mL) was required to kill extracellular bacteria for *E. coli* 13I. This higher concentration did not impair J774 and Caco2 viability or monolayer formation (data not shown). Aliquots of J774 macrophage culture supernatants (500 µL each) at 24 hr post-infection were collected and stored at -20°C until use in a TNF-α ELISA (eBioscience; San Diego, CA) according to the manufacturer's instructions. A protocol similar to that used for the J774 infection assays was also applied to the Caco2 infection assays, except the infection period was 3 hr instead of 2 hr. Also, no gentamycin treatment was applied to cultures of Caco2 cells used to evaluate *E. coli* adherence.

Mouse co-colonization experiments to establish therapeutic potential of *E. coli* strains

Antibiotic susceptibility for *E. coli* strains was first assessed by disc diffusion using antibiotic sensitivity discs (Thermo Fisher Scientific) and standard procedures (11). We confirmed these results by plating *E. coli* cultures on EMB agar plates supplemented with antibiotics: 30 µg/mL oxytetracycline for AIEC 13I (oxytetracycline resistant) and non-AIEC T75 (oxytetracycline sensitive), and 100 µg/mL ampicillin for AIEC UM-146 (ampicillin sensitive) and non-AIEC HM488 (ampicillin resistant).

Bacterial adherence to the cecal epithelium was determined using a previously published method (12) with minor modification. Briefly, cecal tissues were excised, thoroughly washed with 1X PBS, weighed, and resuspended in PBS with 0.1% Triton-X-100 (Thermo Fisher Scientific) to make a 10^{-1} dilution. Tissues were then homogenized using gentleMACS C tubes and program m_spleen_02 on a gentleMACS dissociator (Miltenyi Biotec; CA, USA). Homogenates were serially diluted (10-fold) and plated in 10 µL volumes in triplicate on EMB agar plates to quantify adherent *E.* coli ($\log_{10}$ CFU/g tissue). For co-colonization experiments, cecal tissue homogenates were plated on both regular and antibiotic supplemented EMB agar plates. The CFU count for the antibiotic sensitive *E. coli* strains were calculated as the difference between the total CFU count for both *E. coli* strains and that of the resistant *E. coli* strain.

To quantify luminal levels of *E. coli*, DNA was isolated from cecal contents as previously described using a phenol-chloroform-isoamyl alcohol and chloroform-isoamyl alcohol based protocol (3, 13). Strain-specific primers (13I_3_F: GGCCCAAATGGTGTGAAGTTC; 13I_3_R: GCAGCTTTTGTCACAGCGTTA; UM146_7_F: TACTGGACTTGCTCGTGCTTT; UM146_7_R: TCTGACTCGAACCCCTCATCT; T75_4_F: GATGGCCCGGTAAGTATGGAG; T75_4_R: GTTGCAACAAAGCAGACGACT; HM488_3_F: GTTTGCTGCACTTTTGAACGC; HM488_3_R: CCAGCTCCTTCAGTGAGTTGT) were designed using genomes sequenced on an

Illumina Miseq System (Illumina, San Diego, CA, USA). The raw data and genome assemblies for the four strains are associated with NCBI BioProject PRJNA556430.

In vitro co-colonization experiments

E. coli strains were grown to late-log phase prior to centrifugation and resuspension in LB broth to a density of $1x10^8$ CFU/mL. To create the co-cultures, 100 mL of fresh LB broth was inoculated with $1x10^8$ CFU of each *E. coli* strain. At each time point, 1 mL of broth was sampled from each culture and serially diluted ten-fold. A volume of 10 uL from each dilution was then plated on regular or antibiotic supplemented EMB agar in triplicate prior to aerobic incubation overnight at 37C for enumeration of colonies. The CFU count for the antibiotic sensitive *E. coli* strains was calculated as the difference between the total CFU count for both *E. coli* strains and that of the resistant *E. coli* strain.

For co-infection cell culture experiments, Caco2 and J774 cells were infected with either AIEC 13I, non-AIEC T75 or a combination of both strains at an MOI 5 per strain. Following infection, lysates were serially diluted ten-fold and plated on both regular and antibiotic supplemented EMB agar plates. The CFU count for the antibiotic sensitive *E. coli* strains were calculated as the difference between the total CFU count for both *E. coli* strains and that of the antibiotic resistant *E. coli* strain.

Whole-genome sequencing

E. coli (strains 13I, UM-146, T75 and HM488) were harvested from an overnight culture and genomic DNA was isolated using a DNeasy Blood and Tissue kit (Qiagen®, Germantown, MD) as per manufacturer instructions. Pair-end libraries were prepared using Illumina Nextera® XT DNA Library Prep Kit (Illumina), and quality control was tested by Agilent High Sensitivity

D1000 ScreenTape analysis (Agilent). DNA was quantified using fluorescent molecule labeling, and all the libraries were diluted to a final concentration of 4 nM prior to pooling them together.

Libraries for each genome were prepared and barcoded using the NEBNext Ultra II DNA Library Prep Kit (New England Biolabs). The resulting libraries were pooled and sequenced on the Illumina MiSeq platform (500 cycles, MiSeq Reagent Kit v2) using 250 bp paired-end sequencing. Sequencing adapters were removed with BBDuk of the BBMap software suite (version 37.17, default parameters) (14). VSEARCH was used to remove sequences containing ambiguous bases, reads shorter than 100 bp, or reads containing an estimated error rate greater than 1% (version 2.0.3; parameters: -fastq_maxns 0 -fastq_maxee_rate 0.01 -fastq_minlen 100) (15). Reads were trimmed at low abundant k-mers to further remove potential sequencing errors (Khmer version 2.0) (16). Due to high sequencing depth, paired-end reads were subsampled to ~100X coverage for the T75 strain. Following quality control, paired-end reads for each genome were assembled with Spades (version 3.10.1) using kmer sizes of 21, 33, 55, 77, 99, and 127 and the careful parameter evoked (17). Assembled contigs shorter than 1 kbp were removed. The estimated completeness and contamination of the resulting genomes were assessed with CheckM (18). All genomes were estimated to be >99% complete with <0.5% contamination. The raw data and genome assemblies for the four strains are associated with NCBI BioProject PRJNA556430.

Strain-specific primer design and validation

Genomic sequences of ASF strains were downloaded from GeneBank-NCBI database (http://www.ncbi.nlm.nih.gov/genbank/) and can be identified using the following GenBank accession numbers: *Clostridium sp.* (ASF 356; AQFQ00000000.1), *Lactobacillus intestinalis* (ASF 360; AQFR00000000.1), *Lactobacillus murinus* (ASF 361; AQFS00000000.1), *Mucispirillum schaedleri* (ASF 457; AYGZ00000000.1), *Eubacterium plexicaudatum* (ASF 492;

AQFT00000000.1), *Pseudoflavonifractor sp.* (ASF 500; AYJP00000000.1), *Clostridium sp.* (ASF 502; AQFU00000000.1), *Parabacteroides goldsteinii* (ASF 519; AQFV00000000.1). Putative primer pairs that specifically targeted one *E.coli* strain and not the other three *E.coli* strains or any of the eight ASF members were designed using RUCS-1.0 (https://cge.cbs.dtu.dk/services/RUCS/) (19) with minor modifications of default settings: k-mer size changed to 22 and product size range changed to 200-500. Candidate primers were experimentally validated for specificity and efficiency by real-time quantitative PCR (qPCR) using genomic DNA from the four *E. coli* strains used in mouse co-colonization experiments (strains 13I, UM-146, T75 and HM488) and the eight ASF members.

Real-time quantitative PCR

DNA was isolated from feces and cecal contents as previously described using a phenol-chloroform-isoamyl alcohol and chloroform-isoamyl alcohol-based protocol (3, 13). DNA was quantified using fluorescent molecule labeling, and all samples were diluted to a final concentration of 10 ng/μL prior to using 1 μL of the DNA template in each qPCR reaction as previously described (3). Primer sequences are provided below. All primers were synthesized by Integrated DNA Technologies (Coralville, IA). PCR reactions were prepared as previously described (3), and thermocycling conditions included: (i) an initial denaturation step of 10 min at 95°C; (ii) 35 cycles of 15s at 95°C, 15s at 58°C (annealing temperature), and 20s at 68°C; (iii) one cycle of 15s at 95°C; (iv) one cycle of 15s at 60°C; (v) one 20-min interval to generate a melting curve; and (vi) one cycle of 15s at 95°C.

References

1. Dewhirst FE, Chien CC, Paster BJ, Ericson RL, Orcutt RP, Schauer DB, Fox JG. 1999. Phylogeny of the defined murine microbiota: altered Schaedler flora. Appl Environ Microbiol 65:3287-92.

2. Wymore Brand M, Wannemuehler MJ, Phillips GJ, Proctor A, Overstreet AM, Jergens AE, Orcutt RP, Fox JG. 2015. The altered Schaedler flora: Continued applications of a defined murine microbial community. ILAR J 56:169-78.

3. Gomes-Neto JC, Mantz S, Held K, Sinha R, Segura Munoz RR, Schmaltz R, Benson AK, Walter J, Ramer-Tait AE. 2017. A real-time PCR assay for accurate quantification of the individual members of the Altered Schaedler Flora microbiota in gnotobiotic mice. J Microbiol Methods 135:52-62.

4. Wirth T, Falush D, Lan R, Colles F, Mensa P, Wieler LH, Karch H, Reeves PR, Maiden MC, Ochman H, Achtman M. 2006. Sex and virulence in Escherichia coli: an evolutionary perspective. Mol Microbiol 60:1136-51.

5. Hall TA. 1999. BioEdit: a user-friendly biological sequence alignment editor and analysis program for Windows 95/98/NT. Nucleic Acids Symp Ser (Oxf) 41:95-98.

6. Tamura K, Nei M. 1993. Estimation of the number of nucleotide substitutions in the control region of mitochondrial DNA in humans and chimpanzees. Mol Biol Evol 10:512-26.

7. Kumar S, Stecher G, Tamura K. 2016. MEGA7: Molecular evolutionary genetics analysis version 7.0 for bigger datasets. Mol Biol Evol 33:1870-4.

8. Boudeau J, Glasser AL, Masseret E, Joly B, Darfeuille-Michaud A. 1999. Invasive ability of an Escherichia coli strain isolated from the ileal mucosa of a patient with Crohn's disease. Infect Immun 67:4499-509.

9. Glasser AL, Boudeau J, Barnich N, Perruchot MH, Colombel JF, Darfeuille-Michaud A. 2001. Adherent invasive *Escherichia coli* strains from patients with Crohn's disease survive and replicate within macrophages without inducing host cell death. Infect Immun 69:5529-37.

10. Darfeuille-Michaud A, Boudeau J, Bulois P, Neut C, Glasser AL, Barnich N, Bringer MA, Swidsinski A, Beaugerie L, Colombel JF. 2004. High prevalence of adherent-invasive Escherichia coli associated with ileal mucosa in Crohn's disease. Gastroenterology 127:412-21.

11. Shafi MS. 1975. Determination of antimicrobial MIC by paper diffusion method. J Clin Pathol 28:989-92.

12. Kittana H, Quintero-Villegas MI, Bindels LB, Gomes-Neto JC, Schmaltz RJ, Segura Munoz RR, Cody LA, Moxley RA, Hostetter J, Hutkins RW, Ramer-Tait AE. 2018. Galactooligosaccharide supplementation provides protection against *Citrobacter rodentium*-induced colitis without limiting pathogen burden. Microbiology 164:154-162.

13. Martinez I, Wallace G, Zhang C, Legge R, Benson AK, Carr TP, Moriyama EN, Walter J. 2009. Diet-induced metabolic improvements in a hamster model of hypercholesterolemia are strongly linked to alterations of the gut microbiota. Appl Environ Microbiol 75:4175-84.

14. Bushnell B. BBMap. sourceforge.net/projects/bbmap/.

15. Rognes T, Flouri T, Nichols B, Quince C, Mahe F. 2016. VSEARCH: a versatile open source tool for metagenomics. PeerJ 4:e2584.

16. Crusoe MR, Alameldin HF, Awad S, Boucher E, Caldwell A, Cartwright R, Charbonneau A, Constantinides B, Edvenson G, Fay S, Fenton J, Fenzl T, Fish J, Garcia-Gutierrez L, Garland P, Gluck J, Gonzalez I, Guermond S, Guo J, Gupta A, Herr JR, Howe A, Hyer A, Harpfer A, Irber L, Kidd R, Lin D, Lippi J, Mansour T, McA'Nulty P, McDonald E, Mizzi J, Murray KD, Nahum JR, Nanlohy K, Nederbragt AJ, Ortiz-Zuazaga H, Ory J, Pell J, Pepe-Ranney C, Russ ZN, Schwarz E, Scott C, Seaman J, Sievert S, Simpson J, Skennerton CT, Spencer J, Srinivasan R, Standage D, et al. 2015. The khmer software package: enabling efficient nucleotide sequence analysis. F1000Res 4:900.

17. Bankevich A, Nurk S, Antipov D, Gurevich AA, Dvorkin M, Kulikov AS, Lesin VM, Nikolenko SI, Pham S, Prjibelski AD, Pyshkin AV, Sirotkin AV, Vyahhi N, Tesler G, Alekseyev MA, Pevzner PA. 2012. SPAdes: a new genome assembly algorithm and its applications to single-cell sequencing. J Comput Biol 19:455-77.

18. Parks DH, Imelfort M, Skennerton CT, Hugenholtz P, Tyson GW. 2015. CheckM: assessing the quality of microbial genomes recovered from isolates, single cells, and metagenomes. Genome Res 25:1043-55.

19. Thomsen MCF, Hasman H, Westh H, Kaya H, Lund O. 2017. RUCS: rapid identification of PCR primers for unique core sequences. Bioinformatics 33:3917-3921.
